# A Brief History of the Metropolitan Hospital Sunday Fund

**Published:** 1899-09-16

**Authors:** 


### A BRIEF HISTORY OF THE METROPOLITAN HOSPITAL SUNDAY FUND.

We reproduce the following facts from the tenth issue of Burdett's "Hospitals and Charities," just published, as it cannot fail to interest many readers and hospital authorities everywhere:—

### History of the Fund.

The Metropolitan Hospital Sunday Fund during the 27 years of its existence has raised for the benefit of the hospitals a sum which exceeds £1,000,000 sterling. Sir Sydney Waterlow, who has been connected with the Fund from the outset, and who was mainly instrumental in starting it in London, is therefore to be warmly congratulated on the fulfilment of the hope he recently expressed that he might live to see the amount collected reach this sum. We are sure that everyone connected with hospitals will desire to offer the heartiest congratulations to Sir Sydney Waterlow on the attainment of this magnificent result, which justifies the publication of the following brief summary of the history of the Metropolitan Hospital Sunday Fund.

In 1873, when Sir Sydney Waterlow was Lord Mayor, he communicated with the Lord Mayor of Birmingham (in which town the Hospital Sunday Fund was first established in 1859), asking him for full particulars of the organisation and methods adopted. Sir Sydney's letter was handed by the Lord Mayor of Birmingham to Sir Henry Burdett, and he prepared a statement and supplied all forms, with the aid of which Sir Sydney Waterlow in 1873 instituted the Hospital Sunday Fund in London. In that year only 1,072 congregations contributed, and the total receipts amounted to £27,700. There was an increase of £2,000 in the amount collected in 1874, from which year the total receipts gradually diminished till they reached the minimum of £24,905 in 1878. An increase was then perceptible year by year, till the receipts rose to £39,330 in 1884, when the contributing congregations amounted to 1,522. In 1885 the total receipts fell to £34,320, and in that year Dr. James Wakley, who was suffering from a mortal disease, sent for the writer and urged him to devise some scheme which would help the hospitals by increasing the interest in and collections on Hospital Sunday. After due deliberation a number of public meetings were organised throughout London at which some of the most eminent leaders of public opinion kindly consented to speak. Dr. Wakley, as editor of the *Lancet*, published a special supplement, which was edited by Sir Henry Burdett, and widely circulated. In the result, the total receipts in 1886, although the contributing congregations only amounted to 1,595, reached £40,399.

### The Origin of "The Hospital."

With a view of still further stimulating the interest of the public in the voluntary system of hospital support, the late Dr. Wakley and Sir Henry Burdett discussed together the outlines of a paper which should deal with all the questions involved by the maintenance of an adequate hospital system. In the result, the first number of The Hospital was published on October 2nd, 1886. In the year following Dr. Wakley's death (1887) the proprietors of the *Lancet* discontinued the issue of a special supplement, and The Hospital filled the void thus created by publishing a special supplement, and has continued the practice ever since. In 1888 the *Lancet* again issued a special supplement, and the present proprietors, like The Hospital, have continued to do so, and have circulated many thousands of abstracts from it amongst the congregations each year.

### The Yearly Collections.

Up to and including the year 1892 the total receipts always exceeded £40,000, the maximum being reached in 1891, when they amounted to £45,331, including donations of £5,000 from the late Duke of Cleveland and £1,000 from Sir Savile Crossley, Bart., the latter of whom has continued to give a similar sum annually up to and including the year 1896. In 1893 the total receipts fell to £39,291, and in 1894 to £38,680, exclusive of a legacy of £5,000 bequeathed by the late Mr. W. I. Whitaker. For nearly twenty years Mr. Henry N.

Custance, the present secretary of the Fund, has laboured assiduously and successfully to increase the number of contributing congregations, which now amount to about 1,800, as compared with 1,072 at the outset. By his attention to the requirements of the poor who seek hospital and convalescent aid, and surgical appliances through the agency of the Fund, Mr. Custance has rendered yeoman's service.

The Collection of 1899.

Still, the fact that the amounts received from congregations fell to £35,962 in 1894, the smallest sum received, with one exception, in any year since 1886, induced the proprietors of The Hospital to organise the special effort during the year 1895, which proved so successful that Hospital Sunday in London was lifted out of the rut of £40,000 and increased to £60,000.

This effort was originated by The Hospital newspaper, and was cordially supported by the editors of the metropolitan press, who enthusiastically co-operated to make the effort a success. The Council of the Metropolitan Hospital Sunday Fund in their report stated that nearly 60,000 copies of the special supplement of The Hospital were circulated at the cost of the editor and a few of his friends, and that £18,000 was sent in contributions to the Lord Mayor as the result of The Hospital appeal. This magnificent result of the special efforts of the press raised the Hospital Sunday Fund in London in 1895 to the unprecedented sum of £60,360. Of this sum £38,371 was received from congregations, and £21,990 from legacies and special donations. The last item includes the first dividend from the Court of Chancery on the Guesdon bequest of £45,346 2¾ per cent. Consols, amounting to £904 for nine months; £3,400 from the members of the Stock Exchange; £10,000 from Messrs. B. J. Barnato, David Marks, and about 500 friends; and £1,000 each from Lord Iveagh, Sir Savile Crossley, Bart. (fifth year), and Mr. J. B. Robinson.

The Contributing Congregations.

In 1896 there were 1,811 contributing congregations, the largest number since the Fund was instituted, who collectively raised £40,502 in the churches. Legacies, special donations, and dividends brought the total sum received to £46,035, the largest sum ever received except that contributed during the previous year under the special circumstances already explained In 1897 and 1898 the Fund somewhat languished, the number of contributing congregations falling to 1,785, and the total receipts to about £40,750.

The Diamond Jubilee.

Eighteen hundred and ninety-seven was the Jubilee year, in which the Prince of Wales started his special Fund for hospitals, and many people have thought and said that that Fund must interfere with the Hospital Sunday Fund. That this fear was groundless, however, is proved by the circumstance that during the year 1899 the total receipts are estimated by the Distribution Committee to amount to nearly £53,000, of which upwards of £50,000 was distributed amongst the hospitals. This £50,000 includes a special donation of £10,000 from Mr. George Herring, who has frequently shown the deepest interest in the work of the hospitals, and is one of their most bountiful and valued supporters. Mr. Densham also generously gave 1,000 guineas to the Fund.

The Work of Sir Sydney Waterlow.

Sir Sydney Waterlow has occupied the position of vice-president to the Metropolitan Hospital Sunday Fund from the year of its institution, and has never missed being present at the meeting each year to move the adoption of the report of the Committee of Distribution. The Fund has greatly prospered under his direction, for when the committee began its work in 1874, 17 general hospitals, 6 convalescent homes, and 3 cottage hospitals applied for grants against 31 general hospitals, 29 convalescent institutions, and 14 cottage hospitals asking for help in 1899. These figures are very gratifying, in view of the fact that the population of London increases so largely that a population approaching that of an average sized provincial town is added to the metropolis every year. It is pleasant further to be able to record that increased accommodation for the sick poor has been provided during the years the Fund has existed, which bears some proportion to the growth of population. We offer our sincere congratulations to the successive Lord Mayors of London, to Sir Sydney Waterlow, the vice-president, and to the Council and officers of the Fund on the success which the Fund has achieved under their able administration.

